# Supplementary material for: Belowground fungal community diversity, composition and ecological functionality associated with winter wheat in conventional and organic agricultural systems
Source: PeerJ. 2020 Oct 13;8:e9732. doi: 10.7717/peerj.9732 (PMC7566770; doi:10.7717/peerj.9732)
Supplement: Supplemental Information 1 [file peerj-08-9732-s001.docx]

| **Site** | **Edge A** | **Central area** | **Edge J** |
| --- | --- | --- | --- |
| **C1** | Frequent: *Poa annua, Veronica persica* |  | Frequent*: Poa annua* |
|  | Occasional: *Capsella bursa-pastoris, Galium aparine, Plantago lanceolata, Taraxacum officinale* | Very rarely: *Taraxacum officinale* | Occasional*: Poa trivialis, Taraxacum officinale, Vicia* sp. |
| **C2** | Frequent: *Poa annua* |  | Frequent: *Alopecurus pratensis*, *Poa pratensis* |
|  | Occasional: *Convolvolus arvensis, Plantago media, Potentilla anserina* | Occasional: *Poa trivialis*, *Rumex* sp., *Veronica hederifolia* | Occasional: *Plantago lanceolata* |
| **C3** | Frequent: *Alopecurus pratensis, Erophila verna, Galium mollugo*, *Veronica persica*, remains of previously growing maize | Frequent: *Alopecurus pratensis*, *Veronica hederifolia* | Frequent: *Cardaria draba*, *Dactylis glomerata, Galium mollugo*, *Geranium dissectum,* Neighbor field: *Hordeum vulgare* |
|  | Occasional: *Veronica hederifolia, Viola arvensis* | Occasional: *Galium mollugo* | Occasional: *Convolvolus arvensis*, *Thlaspi arvense* |
| **O1** | Frequent: *Veronica persica, Galium mollugo, Cardaria draba* | Frequent: *Capsella bursa-pastoris, Lamium purpureum*, *Veronica persica, Vicia* sp. | Frequent: *Galium mollugo, Geum urbanum, Dactylis glomerata, Medicago sativa, Potentilla reptans, Veronica persica, Vicia* sp. |
|  | Occasional: *Brassica napus, Crataegus monogyna, Geranium pratense, Geum urbanum, Prunus spinosa, Rosa canina, Rumex* sp. | Occasional: *Brassica napus*, *Bromus hordaceus, Myosotis arvensis* | Occasional:  *Cardaria draba, Falcaria vulgaris, Galium aparine, Knautia arvensis, Poa trivialis, Thlaspi arvense* |
| **O2** | Frequent: *Brassica napus, Capsella bursa-pastoris, Veronica persica* | Frequent: *Brassica napus, Medicago sativa, Rumex* sp*., Galium aparine, Lamium purpureum, Veronica persica* | Frequent: *Brassica napus*, *Dactylis glomerata, Galium aparine, Poa trivialis, Veronica persica* |
|  | Occasional: *Convolvolus arvensis, Lamium purpureum* | Occasional: *Fumaria officinalis, Taraxacum officinalis* | Occasional: *Geranium pratense, Medicago sativa* |
| **O3** | Frequent: *Brassica napus, Lamium purpureum, Medicago sativa, Veronica persica* | Frequent: *Brassica napus, Galium aparine, Lamium purpureum*, *Medicago sativa, Rumex* sp*.* | Frequent: *Brassica napus, Corylus avellana, Galium aparine, Salix rubens*, *Tilia* sp., *Urtica dioica* |
|  | Occasional: *Capsella bursa-pastoris, Convolvolus arvensis, Plantago media* |  | Occasional: *Anthriscus sylvestris*, *Ranunculus acris, Ranunculus ficcaria, Rumex* sp., *Taraxacum officinale* |
