# Supplementary material for: Belowground fungal community diversity, composition and ecological functionality associated with winter wheat in conventional and organic agricultural systems
Source: PeerJ. 2020 Oct 13;8:e9732. doi: 10.7717/peerj.9732 (PMC7566770; doi:10.7717/peerj.9732)
Supplement: Supplemental Information 2 [file peerj-08-9732-s002.docx]

| **Herbarium**  **Number** | **Farming system** | **Plot** | **ITS1/ITS4 direct sequencing** | **ITS1/ITS4 sequencing after cloning** | **NSI1/NL4 direct sequencing** | **NSI1/NL4 sequencing after cloning** |
| --- | --- | --- | --- | --- | --- | --- |
| TUB 021520 | C1 | B |  |  |  | KY430531  KY430533  KY430473 KY430476  KY430477 |
| TUB 021523 | C1 | E | KY430469 KY430493 |  |  |  |
| TUB 021522 | C1 | G |  | KY430446 KY430454 KY430455 KY430467  KY430501 |  |  |
| TUB 021525 | C1 | I | KY430503 |  |  |  |
| TUB 021529 | C2 | A |  |  |  | KY430535 |
| TUB 021533 | C2 | E | KY430461 | KY430506 | KY430507 |  |
| TUB 021534 | C2 | F | KY430460 | KY430458 KY430505 | KY430526 | KY430525 KY430523 KY430524 |
| TUB 021535 | C2 | G |  |  | KY430480 |  |
| TUB 021536 | C2 | H |  |  |  | KY430508 |
| TUB 021529 | C2 | J | KY430492 |  |  |  |
| TUB 021540 | C3 | B |  |  | KY430522 |  |
| TUB 021542 | C3 | D | KY430470 |  |  |  |
| TUB 021544 | C3 | F |  | KY430459 |  |  |
| TUB 021545 | C3 | G |  |  | KY430534 |  |
| TUB 021546 | C3 | H |  | KY430497 |  |  |
| TUB 021547 | C3 | I |  | KY430466 |  |  |
| TUB 021548 | C3 | J |  | KY430500 KY430451 |  | KY430512 |
| TUB 021580 | C4 | B |  | KY430566 KX430539  KY430560 KY430567 KY430561 KY430565 |  |  |
| TUB 021581 | C4 | C | KY430541 |  |  |  |
| TUB 021586 | C4 | H |  | KY430550 KY430553 |  |  |
| TUB 021589 | C5 | A |  | KY430568 KY430558 KY430559 KY430569 |  |  |
| TUB 021591 | C5 | C | KY430570, KY430564 |  |  |  |
| TUB 021592 | C5 | D |  | KY430571 KY430572 KY430563 KY430573 KY430574 |  |  |
| TUB 021593 | C5 | E | KY430575, KY430549 |  |  |  |
| TUB 021594 | C5 | F |  | KY430540 KY430554 |  |  |
| TUB 021595 | C5 | G |  | KY430551 KY430556 KY430557 KY430576 KY430577 KY430578 |  |  |
| TUB 021596 | C5 | H |  | KY430562 KY430579 KY430547 KY430548 |  |  |
| TUB 021598 | C5 | J |  | KY430580 KY430581 KY430582 KY430583 |  |  |
| TUB 021551 | O1 | C | KY430456 |  |  |  |
| TUB 021552 | O1 | D |  |  |  | KY430527 |
| TUB 021553 | O1 | E |  | KY430468 KY430499 |  |  |
| TUB 021554 | O1 | F | KY430504 |  |  | KY430532 KY430472 KY430478 KY430479 KY430511 |
| TUB 021555 | O1 | G |  |  |  | KY430513 KY430537 |
| TUB 021556 | O1 | H |  |  |  | KY430536 |
| TUB 021557 | O1 | I |  | KY430502 KY430464 KY430465 KY430498 |  |  |
| TUB 021558 | O1 | J |  |  | KY430521 |  |
| TUB 021559 | O2 | A |  | KY430457 |  |  |
| TUB 021560 | O2 | B |  | KY430496 |  |  |
| TUB 021562 | O2 | D |  |  |  | KY430584 |
| TUB 021563 | O2 | E |  | KY430453 KY430489 KY430490 KY430491 |  |  |
| TUB 021564 | O2 | F | KY430488 |  |  |  |
| TUB 021565 | O2 | G |  | KY430495 KY430447 KY430448 |  |  |
| TUB 021566 | O2 | H |  | KY430462 KY430487 KY430494 | KY430520 |  |
| TUB 021567 | O2 | I |  | KY430484 KY430485 KY430486 KY430449 KY430450 KY430452 KY430463 |  | KY430475 KY430519 |
| TUB 021568 | O2 | J |  | KY430483 |  | KY430481 KY430509 |
| TUB 021569 | O3 | A |  |  | KY430514 |  |
| TUB 021573 | O3 | E |  | KY430482 |  |  |
| TUB 021575 | O3 | G |  |  |  | KY430517 KY430518 KY430528 KY430529 KY430530 KY430538 KY430471 |
| TUB 021576 | O3 | H |  |  | KY430516 |  |
| TUB 021577 | O3 | I |  |  |  | KY430474 KY430510 KY430515 |
| TUB 021609 | O4 | A | KY430545 |  |  |  |
| TUB 021610 | O4 | B | KY430543 |  |  |  |
| TUB 021616 | O4 | H | KY430542 |  |  |  |
| TUB 021622 | O5 | D |  | KY430544  KY430546 KY430555 KY430552 |  |  |
