# Supplementary material for: Belowground fungal community diversity, composition and ecological functionality associated with winter wheat in conventional and organic agricultural systems
Source: PeerJ. 2020 Oct 13;8:e9732. doi: 10.7717/peerj.9732 (PMC7566770; doi:10.7717/peerj.9732)
Supplement: Supplemental Information 3 [file peerj-08-9732-s003.docx]

| **Farming system** | **pHCaCl2** | **CaCO3 [%]** | **Nt [%]** | **Ct [%]** | **St [%]** | **Corg [%]** | **Kcal [mg/kg]** | **Pcal [mg/kg]** | **C/N ratio** | **Humus [%]** |
| --- | --- | --- | --- | --- | --- | --- | --- | --- | --- | --- |
| C1 | 6.175 |  | 0.17 | 1.585 | 0.035 | 1.585 | 215.89 | 53.255 | 9.36 | 2.725 |
| C2 | 6.84 | 1.46 | 0.175 | 1.775 | 0.035 | 1.69 | 190.025 | 61.225 | 10.03 | 2.905 |
| C3 | 7.23 | 9.355 | 0.235 | 2.905 | 0.035 | 1.785 | 443.78 | 127.515 | 12.545 | 3.065 |
| C4 | 6.65 | 1.23 | 0.16 | 1.51 | 0.035 | 1.435 | 222.77 | 41.23 | 9.37 | 2.465 |
| C5 | 7.24 | 9.72 | 0.24 | 3.06 | 0.04 | 1.89 | 479.645 | 126.715 | 12.73 | 3.25 |
| O1 | 7.28 | 14.18 | 0.205 | 3.725 | 0.04 | 2.02 | 235.455 | 135.245 | 19.04 | 3.475 |
| O2 | 7.275 | 15.1 | 0.21 | 4.055 | 0.04 | 2.245 | 252.785 | 107.09 | 19.17 | 3.86 |
| O3 | 7.37 | 10.885 | 0.255 | 3.87 | 0.045 | 2.565 | 156.24 | 142.58 | 15.12 | 4.41 |
| O4 | 7.21 | 10.47 | 0.225 | 3.69 | 0.035 | 2.435 | 332.31 | 243.865 | 16.86 | 4.19 |
| O5 | 7.425 | 7.855 | 0.22 | 3.42 | 0.045 | 2.475 | 132.95 | 133.41 | 15.345 | 4.255 |
