# Supplementary material for: Belowground fungal community diversity, composition and ecological functionality associated with winter wheat in conventional and organic agricultural systems
Source: PeerJ. 2020 Oct 13;8:e9732. doi: 10.7717/peerj.9732 (PMC7566770; doi:10.7717/peerj.9732)
Supplement: Supplemental Information 4 — Extended results for Table 1 [file peerj-08-9732-s004.pdf]

Table 1: Test

	sequence ID	MOTU	taxonomy	species	guild	trophic mode	farming system	agricultural field	plot	taxonomy path
1	KY430446	7	159639	Helotiales incertae sedis	-	-	Conventional	C1	G	root; cellular organisms; [SK] Eukaryota; Opisthokonta; [K] Fungi; Dikarya; [P] Ascomycota; saccharomyceta; Pezizomycotina; leotiomyceta; sordariomyceta; [C] Leotiomycetes; [O] Helotiales; Helotiales incertae sedis;
2	KY430447	7	4890	Ascomycota	-	-	Organic	O2	G	root; cellular organisms; [SK] Eukaryota; Opisthokonta; [K] Fungi; Dikarya; [P] Ascomycota;
3	KY430448	7	4890	Ascomycota	-	-	Organic	O2	G	root; cellular organisms; [SK] Eukaryota; Opisthokonta; [K] Fungi; Dikarya; [P] Ascomycota;
4	KY430449	7	4890	Ascomycota	-	-	Organic	O2	I	root; cellular organisms; [SK] Eukaryota; Opisthokonta; [K] Fungi; Dikarya; [P] Ascomycota;
5	KY430451	19	252155	Schizothecium	Dung Saprotroph	Saprotroph	Conventional	C3	J	root; cellular organisms; [SK] Eukaryota; Opisthokonta; [K] Fungi; Dikarya; [P] Ascomycota; saccharomyceta; Pezizomycotina; leotiomyceta; sordariomyceta; [C] Sordariomycetes; Sordariomycetidae; [O] Sordariales; [F] Lasiosphaeriaceae; [G] Schizothecium;
6	KY430452	38	39397	Candida sake	Undefined Saprotroph	Saprotroph	Organic	O2	I	root; cellular organisms; [SK] Eukaryota; Opisthokonta; [K] Fungi; Dikarya; [P] Ascomycota; saccharomyceta; Saccharomycotina; [C] Saccharomycetes; [O] Saccharomycetales; Saccharomycetales incertae sedis; [G] Candida; [S] Candida sake;
7	KY430453	38	39397	Candida sake	Undefined Saprotroph	Saprotroph	Organic	O2	E	root; cellular organisms; [SK] Eukaryota; Opisthokonta; [K] Fungi; Dikarya; [P] Ascomycota; saccharomyceta; Saccharomycotina; [C] Saccharomycetes; [O] Saccharomycetales; Saccharomycetales incertae sedis; [G] Candida; [S] Candida sake;
8	KY430454	27	715340	Pleosporineae	-	-	Conventional	C1	G	root; cellular organisms; [SK] Eukaryota; Opisthokonta; [K] Fungi; Dikarya; [P] Ascomycota; saccharomyceta; Pezizomycotina; leotiomyceta; dothideomyceta; [C] Dothideomycetes; Pleosporomycetidae; [O] Pleosporales; Pleosporineae;
9	KY430455	29	683158	Didymellaceae	Animal Pathogen-Plant Pathogen-Undefined Saprotroph	Pathotroph-Saprotroph	Conventional	C1	G	root; cellular organisms; [SK] Eukaryota; Opisthokonta; [K] Fungi; Dikarya; [P] Ascomycota; saccharomyceta; Pezizomycotina; leotiomyceta; dothideomyceta; [C] Dothideomycetes; Pleosporomycetidae; [O] Pleosporales; Pleosporineae; [F] Didymellaceae;
10	KY430456	28	715340	Pleosporineae	-	-	Organic	O1	C	root; cellular organisms; [SK] Eukaryota; Opisthokonta; [K] Fungi; Dikarya; [P] Ascomycota; saccharomyceta; Pezizomycotina; leotiomyceta; dothideomyceta; [C] Dothideomycetes; Pleosporomycetidae; [O] Pleosporales; Pleosporineae;
11	KY430457	20	67608	Microdochium	Endophyte-Plant Pathogen	Pathotroph-Symbiotroph	Organic	O2	A	root; cellular organisms; [SK] Eukaryota; Opisthokonta; [K] Fungi; Dikarya; [P] Ascomycota; saccharomyceta; Pezizomycotina; leotiomyceta; sordariomyceta; [C] Sordariomycetes; Xylariomycetidae; [O] Xylariales; [F] Microdochiaceae; [G] Microdochium;
12	KY430458	20	67608	Microdochium	Endophyte-Plant Pathogen	Pathotroph-Symbiotroph	Conventional	C2	F	root; cellular organisms; [SK] Eukaryota; Opisthokonta; [K] Fungi; Dikarya; [P] Ascomycota; saccharomyceta; Pezizomycotina; leotiomyceta; sordariomyceta; [C] Sordariomycetes; Xylariomycetidae; [O] Xylariales; [F] Microdochiaceae; [G] Microdochium;
13	KY430459	20	67608	Microdochium	Endophyte-Plant Pathogen	Pathotroph-Symbiotroph	Conventional	C3	F	root; cellular organisms; [SK] Eukaryota; Opisthokonta; [K] Fungi; Dikarya; [P] Ascomycota; saccharomyceta; Pezizomycotina; leotiomyceta; sordariomyceta; [C] Sordariomycetes; Xylariomycetidae; [O] Xylariales; [F] Microdochiaceae; [G] Microdochium;
14	KY430460	20	67608	Microdochium	Endophyte-Plant Pathogen	Pathotroph-Symbiotroph	Conventional	C2	F	root; cellular organisms; [SK] Eukaryota; Opisthokonta; [K] Fungi; Dikarya; [P] Ascomycota; saccharomyceta; Pezizomycotina; leotiomyceta; sordariomyceta; [C] Sordariomycetes; Xylariomycetidae; [O] Xylariales; [F] Microdochiaceae; [G] Microdochium;
15	KY430461	20	67608	Microdochium	Endophyte-Plant Pathogen	Pathotroph-Symbiotroph	Conventional	C2	E	root; cellular organisms; [SK] Eukaryota; Opisthokonta; [K] Fungi; Dikarya; [P] Ascomycota; saccharomyceta; Pezizomycotina; leotiomyceta; sordariomyceta; [C] Sordariomycetes; Xylariomycetidae; [O] Xylariales; [F] Microdochiaceae; [G] Microdochium;
16	KY430462	20	67608	Microdochium	Endophyte-Plant Pathogen	Pathotroph-Symbiotroph	Organic	O2	H	root; cellular organisms; [SK] Eukaryota; Opisthokonta; [K] Fungi; Dikarya; [P] Ascomycota; saccharomyceta; Pezizomycotina; leotiomyceta; sordariomyceta; [C] Sordariomycetes; Xylariomycetidae; [O] Xylariales; [F] Microdochiaceae; [G] Microdochium;
17	KY430463	35	1895944	Vishniacozyma victoriae	Fungal Parasite-Undefined Saprotroph	Pathotroph-Saprotroph-Symbiotroph	Organic	O2	I	root; cellular organisms; [SK] Eukaryota; Opisthokonta; [K] Fungi; Dikarya; [P] Basidiomycota; Agaricomycotina; [C] Tremellomycetes; [O] Tremellales; [F] Bulleribasidiaceae; [G] Vishniacozyma; [S] Vishniacozyma victoriae;
18	KY430465	6	155619	Agaricomycetes	-	-	Organic	O1	I	root; cellular organisms; [SK] Eukaryota; Opisthokonta; [K] Fungi; Dikarya; [P] Basidiomycota; Agaricomycotina; [C] Agaricomycetes;
19	KY430466	35	1895944	Vishniacozyma victoriae	Fungal Parasite-Undefined Saprotroph	Pathotroph-Saprotroph-Symbiotroph	Conventional	C3	I	root; cellular organisms; [SK] Eukaryota; Opisthokonta; [K] Fungi; Dikarya; [P] Basidiomycota; Agaricomycotina; [C] Tremellomycetes; [O] Tremellales; [F] Bulleribasidiaceae; [G] Vishniacozyma; [S] Vishniacozyma victoriae;
20	KY430467	5	5209	Filobasidium	Undefined Saprotroph	Saprotroph	Conventional	C1	G	root; cellular organisms; [SK] Eukaryota; Opisthokonta; [K] Fungi; Dikarya; [P] Basidiomycota; Agaricomycotina; [C] Tremellomycetes; [O] Filobasidiales; [F] Filobasidiaceae; [G] Filobasidium;
21	KY430468	5	5209	Filobasidium	Undefined Saprotroph	Saprotroph	Organic	O1	E	root; cellular organisms; [SK] Eukaryota; Opisthokonta; [K] Fungi; Dikarya; [P] Basidiomycota; Agaricomycotina; [C] Tremellomycetes; [O] Filobasidiales; [F] Filobasidiaceae; [G] Filobasidium;
22	KY430469	20	67608	Microdochium	Endophyte-Plant Pathogen	Pathotroph-Symbiotroph	Conventional	C1	E	root; cellular organisms; [SK] Eukaryota; Opisthokonta; [K] Fungi; Dikarya; [P] Ascomycota; saccharomyceta; Pezizomycotina; leotiomyceta; sordariomyceta; [C] Sordariomycetes; Xylariomycetidae; [O] Xylariales; [F] Microdochiaceae; [G] Microdochium;
23	KY430470	20	67608	Microdochium	Endophyte-Plant Pathogen	Pathotroph-Symbiotroph	Conventional	C3	D	root; cellular organisms; [SK] Eukaryota; Opisthokonta; [K] Fungi; Dikarya; [P] Ascomycota; saccharomyceta; Pezizomycotina; leotiomyceta; sordariomyceta; [C] Sordariomycetes; Xylariomycetidae; [O] Xylariales; [F] Microdochiaceae; [G] Microdochium;
24	KY430471	7	4890	Ascomycota	-	-	Organic	O3	G	root; cellular organisms; [SK] Eukaryota; Opisthokonta; [K] Fungi; Dikarya; [P] Ascomycota;
25	KY430472	31	1621986	Darksidea	Endophyte	Symbiotroph	Organic	O1	F	root; cellular organisms; [SK] Eukaryota; Opisthokonta; [K] Fungi; Dikarya; [P] Ascomycota; saccharomyceta; Pezizomycotina; leotiomyceta; dothideomyceta; [C] Dothideomycetes; Pleosporomycetidae; [O] Pleosporales; Massarineae; [F] Lentitheciaceae; [G] Darksidea;
26	KY430473	26	61235	Fusarium equiseti	Animal Pathogen-Endophyte-Lichen Parasite-Plant Pathogen-Soil Saprotroph-Wood Saprotroph	Pathotroph-Saprotroph-Symbiotroph	Conventional	C1	B	root; cellular organisms; [SK] Eukaryota; Opisthokonta; [K] Fungi; Dikarya; [P] Ascomycota; saccharomyceta; Pezizomycotina; leotiomyceta; sordariomyceta; [C] Sordariomycetes; Hypocreomycetidae; [O] Hypocreales; [F] Nectriaceae; [G] Fusarium; Fusarium incarnatum-equiseti species complex; [S] Fusarium equiseti;
27	KY430474	36	4890	Ascomycota	-	-	Organic	O3	I	root; cellular organisms; [SK] Eukaryota; Opisthokonta; [K] Fungi; Dikarya; [P] Ascomycota;
28	KY430475	20	67608	Microdochium	Endophyte-Plant Pathogen	Pathotroph-Symbiotroph	Organic	O2	I	root; cellular organisms; [SK] Eukaryota; Opisthokonta; [K] Fungi; Dikarya; [P] Ascomycota; saccharomyceta; Pezizomycotina; leotiomyceta; sordariomyceta; [C] Sordariomycetes; Xylariomycetidae; [O] Xylariales; [F] Microdochiaceae; [G] Microdochium;
29	KY430476	26	61235	Fusarium equiseti	Animal Pathogen-Endophyte-Lichen Parasite-Plant Pathogen-Soil Saprotroph-Wood Saprotroph	Pathotroph-Saprotroph-Symbiotroph	Conventional	C1	B	root; cellular organisms; [SK] Eukaryota; Opisthokonta; [K] Fungi; Dikarya; [P] Ascomycota; saccharomyceta; Pezizomycotina; leotiomyceta; sordariomyceta; [C] Sordariomycetes; Hypocreomycetidae; [O] Hypocreales; [F] Nectriaceae; [G] Fusarium; Fusarium incarnatum-equiseti species complex; [S] Fusarium equiseti;
30	KY430477	26	61235	Fusarium equiseti	Animal Pathogen-Endophyte-Lichen Parasite-Plant Pathogen-Soil Saprotroph-Wood Saprotroph	Pathotroph-Saprotroph-Symbiotroph	Conventional	C1	B	root; cellular organisms; [SK] Eukaryota; Opisthokonta; [K] Fungi; Dikarya; [P] Ascomycota; saccharomyceta; Pezizomycotina; leotiomyceta; sordariomyceta; [C] Sordariomycetes; Hypocreomycetidae; [O] Hypocreales; [F] Nectriaceae; [G] Fusarium; Fusarium incarnatum-equiseti species complex; [S] Fusarium equiseti;
31	KY430478	33	5144	Podospora	Dung Saprotroph-Endophyte-Litter Saprotroph-Undefined Saprotroph	Saprotroph-Symbiotroph	Organic	O1	F	root; cellular organisms; [SK] Eukaryota; Opisthokonta; [K] Fungi; Dikarya; [P] Ascomycota; saccharomyceta; Pezizomycotina; leotiomyceta; sordariomyceta; [C] Sordariomycetes; Sordariomycetidae; [O] Sordariales; [F] Lasiosphaeriaceae; [G] Podospora;
32	KY430479	8	89926	Cystofilobasidium macerans	Leaf Saprotroph	Saprotroph	Organic	O1	F	root; cellular organisms; [SK] Eukaryota; Opisthokonta; [K] Fungi; Dikarya; [P] Basidiomycota; Agaricomycotina; [C] Tremellomycetes; [O] Cystofilobasidiales; [F] Cystofilobasidiaceae; [G] Cystofilobasidium; [S] Cystofilobasidium macerans;
33	KY430480	20	67608	Microdochium	Endophyte-Plant Pathogen	Pathotroph-Symbiotroph	Conventional	C2	G	root; cellular organisms; [SK] Eukaryota; Opisthokonta; [K] Fungi; Dikarya; [P] Ascomycota; saccharomyceta; Pezizomycotina; leotiomyceta; sordariomyceta; [C] Sordariomycetes; Xylariomycetidae; [O] Xylariales; [F] Microdochiaceae; [G] Microdochium;
34	KY430481	4	5303	Polyporales	-	-	Organic	O2	J	root; cellular organisms; [SK] Eukaryota; Opisthokonta; [K] Fungi; Dikarya; [P] Basidiomycota; Agaricomycotina; [C] Agaricomycetes; Agaricomycetes incertae sedis; [O] Polyporales;
35	KY430482	11	451869	Botryosphaeriales	-	-	Organic	O3	E	root; cellular organisms; [SK] Eukaryota; Opisthokonta; [K] Fungi; Dikarya; [P] Ascomycota; saccharomyceta; Pezizomycotina; leotiomyceta; dothideomyceta; [C] Dothideomycetes; Dothideomycetes incertae sedis; [O] Botryosphaeriales;
36	KY430483	13	715298	Lentitheciaceae	-	-	Organic	O2	J	root; cellular organisms; [SK] Eukaryota; Opisthokonta; [K] Fungi; Dikarya; [P] Ascomycota; saccharomyceta; Pezizomycotina; leotiomyceta; dothideomyceta; [C] Dothideomycetes; Pleosporomycetidae; [O] Pleosporales; Massarineae; [F] Lentitheciaceae;
37	KY430484	5	5209	Filobasidium	Undefined Saprotroph	Saprotroph	Organic	O2	I	root; cellular organisms; [SK] Eukaryota; Opisthokonta; [K] Fungi; Dikarya; [P] Basidiomycota; Agaricomycotina; [C] Tremellomycetes; [O] Filobasidiales; [F] Filobasidiaceae; [G] Filobasidium;
38	KY430485	7	4890	Ascomycota	-	-	Organic	O2	I	root; cellular organisms; [SK] Eukaryota; Opisthokonta; [K] Fungi; Dikarya; [P] Ascomycota;
39	KY430487	24	147548	Leotiomycetes	-	-	Organic	O2	H	root; cellular organisms; [SK] Eukaryota; Opisthokonta; [K] Fungi; Dikarya; [P] Ascomycota; saccharomyceta; Pezizomycotina; leotiomyceta; sordariomyceta; [C] Leotiomycetes;
40	KY430488	7	4890	Ascomycota	-	-	Organic	O2	F	root; cellular organisms; [SK] Eukaryota; Opisthokonta; [K] Fungi; Dikarya; [P] Ascomycota;
41	KY430489	30	4890	Ascomycota	-	-	Organic	O2	E	root; cellular organisms; [SK] Eukaryota; Opisthokonta; [K] Fungi; Dikarya; [P] Ascomycota;
42	KY430490	15	92863	Helminthosporium velutinum	Fungal Parasite-Plant Pathogen	Pathotroph	Organic	O2	E	root; cellular organisms; [SK] Eukaryota; Opisthokonta; [K] Fungi; Dikarya; [P] Ascomycota; saccharomyceta; Pezizomycotina; leotiomyceta; dothideomyceta; [C] Dothideomycetes; Pleosporomycetidae; [O] Pleosporales; Massarineae; [F] Massarinaceae; [G] Helminthosporium; [S] Helminthosporium velutinum;
43	KY430491	34	151289	Vishniacozyma tephrensis	Fungal Parasite-Undefined Saprotroph	Pathotroph-Saprotroph-Symbiotroph	Organic	O2	E	root; cellular organisms; [SK] Eukaryota; Opisthokonta; [K] Fungi; Dikarya; [P] Basidiomycota; Agaricomycotina; [C] Tremellomycetes; [O] Tremellales; [F] Bulleribasidiaceae; [G] Vishniacozyma; [S] Vishniacozyma tephrensis;
44	KY430492	7	4890	Ascomycota	-	-	Conventional	C2	J	root; cellular organisms; [SK] Eukaryota; Opisthokonta; [K] Fungi; Dikarya; [P] Ascomycota;
45	KY430493	20	67608	Microdochium	Endophyte-Plant Pathogen	Pathotroph-Symbiotroph	Conventional	C1	E	root; cellular organisms; [SK] Eukaryota; Opisthokonta; [K] Fungi; Dikarya; [P] Ascomycota; saccharomyceta; Pezizomycotina; leotiomyceta; sordariomyceta; [C] Sordariomycetes; Xylariomycetidae; [O] Xylariales; [F] Microdochiaceae; [G] Microdochium;
46	KY430494	20	67608	Microdochium	Endophyte-Plant Pathogen	Pathotroph-Symbiotroph	Organic	O2	H	root; cellular organisms; [SK] Eukaryota; Opisthokonta; [K] Fungi; Dikarya; [P] Ascomycota; saccharomyceta; Pezizomycotina; leotiomyceta; sordariomyceta; [C] Sordariomycetes; Xylariomycetidae; [O] Xylariales; [F] Microdochiaceae; [G] Microdochium;
47	KY430496	7	4890	Ascomycota	-	-	Organic	O2	B	root; cellular organisms; [SK] Eukaryota; Opisthokonta; [K] Fungi; Dikarya; [P] Ascomycota;
48	KY430497	20	67608	Microdochium	Endophyte-Plant Pathogen	Pathotroph-Symbiotroph	Conventional	C3	H	root; cellular organisms; [SK] Eukaryota; Opisthokonta; [K] Fungi; Dikarya; [P] Ascomycota; saccharomyceta; Pezizomycotina; leotiomyceta; sordariomyceta; [C] Sordariomycetes; Xylariomycetidae; [O] Xylariales; [F] Microdochiaceae; [G] Microdochium;
49	KY430498	14	27381	Funneliformis mosseae	Arbuscular Mycorrhizal	Symbiotroph	Organic	O1	I	root; cellular organisms; [SK] Eukaryota; Opisthokonta; [K] Fungi; [P] Mucoromycota; Glomeromycotina; [C] Glomeromycetes; [O] Glomerales; [F] Glomeraceae; [G] Funneliformis; [S] Funneliformis mosseae;
50	KY430499	7	4890	Ascomycota	-	-	Organic	O1	E	root; cellular organisms; [SK] Eukaryota; Opisthokonta; [K] Fungi; Dikarya; [P] Ascomycota;
51	KY430500	35	1895944	Vishniacozyma victoriae	Fungal Parasite-Undefined Saprotroph	Pathotroph-Saprotroph-Symbiotroph	Conventional	C3	J	root; cellular organisms; [SK] Eukaryota; Opisthokonta; [K] Fungi; Dikarya; [P] Basidiomycota; Agaricomycotina; [C] Tremellomycetes; [O] Tremellales; [F] Bulleribasidiaceae; [G] Vishniacozyma; [S] Vishniacozyma victoriae;
52	KY430501	7	4890	Ascomycota	-	-	Conventional	C1	G	root; cellular organisms; [SK] Eukaryota; Opisthokonta; [K] Fungi; Dikarya; [P] Ascomycota;
53	KY430502	7	4890	Ascomycota	-	-	Organic	O1	I	root; cellular organisms; [SK] Eukaryota; Opisthokonta; [K] Fungi; Dikarya; [P] Ascomycota;
54	KY430503	20	67608	Microdochium	Endophyte-Plant Pathogen	Pathotroph-Symbiotroph	Conventional	C1	I	root; cellular organisms; [SK] Eukaryota; Opisthokonta; [K] Fungi; Dikarya; [P] Ascomycota; saccharomyceta; Pezizomycotina; leotiomyceta; sordariomyceta; [C] Sordariomycetes; Xylariomycetidae; [O] Xylariales; [F] Microdochiaceae; [G] Microdochium;
55	KY430504	7	4890	Ascomycota	-	-	Organic	O1	F	root; cellular organisms; [SK] Eukaryota; Opisthokonta; [K] Fungi; Dikarya; [P] Ascomycota;
56	KY430505	20	67608	Microdochium	Endophyte-Plant Pathogen	Pathotroph-Symbiotroph	Conventional	C2	F	root; cellular organisms; [SK] Eukaryota; Opisthokonta; [K] Fungi; Dikarya; [P] Ascomycota; saccharomyceta; Pezizomycotina; leotiomyceta; sordariomyceta; [C] Sordariomycetes; Xylariomycetidae; [O] Xylariales; [F] Microdochiaceae; [G] Microdochium;
57	KY430506	20	67608	Microdochium	Endophyte-Plant Pathogen	Pathotroph-Symbiotroph	Conventional	C2	E	root; cellular organisms; [SK] Eukaryota; Opisthokonta; [K] Fungi; Dikarya; [P] Ascomycota; saccharomyceta; Pezizomycotina; leotiomyceta; sordariomyceta; [C] Sordariomycetes; Xylariomycetidae; [O] Xylariales; [F] Microdochiaceae; [G] Microdochium;
58	KY430507	20	67608	Microdochium	Endophyte-Plant Pathogen	Pathotroph-Symbiotroph	Conventional	C2	E	root; cellular organisms; [SK] Eukaryota; Opisthokonta; [K] Fungi; Dikarya; [P] Ascomycota; saccharomyceta; Pezizomycotina; leotiomyceta; sordariomyceta; [C] Sordariomycetes; Xylariomycetidae; [O] Xylariales; [F] Microdochiaceae; [G] Microdochium;
59	KY430508	20	67608	Microdochium	Endophyte-Plant Pathogen	Pathotroph-Symbiotroph	Conventional	C2	H	root; cellular organisms; [SK] Eukaryota; Opisthokonta; [K] Fungi; Dikarya; [P] Ascomycota; saccharomyceta; Pezizomycotina; leotiomyceta; sordariomyceta; [C] Sordariomycetes; Xylariomycetidae; [O] Xylariales; [F] Microdochiaceae; [G] Microdochium;
60	KY430509	12	1577607	Slopeiomyces cylindrosporus	Plant Pathogen	Pathotroph	Organic	O2	J	root; cellular organisms; [SK] Eukaryota; Opisthokonta; [K] Fungi; Dikarya; [P] Ascomycota; saccharomyceta; Pezizomycotina; leotiomyceta; sordariomyceta; [C] Sordariomycetes; Sordariomycetidae; [O] Magnaporthales; [F] Magnaporthaceae; [G] Slopeiomyces; [S] Slopeiomyces cylindrosporus;
61	KY430510	22	67608	Microdochium	Endophyte-Plant Pathogen	Pathotroph-Symbiotroph	Organic	O3	I	root; cellular organisms; [SK] Eukaryota; Opisthokonta; [K] Fungi; Dikarya; [P] Ascomycota; saccharomyceta; Pezizomycotina; leotiomyceta; sordariomyceta; [C] Sordariomycetes; Xylariomycetidae; [O] Xylariales; [F] Microdochiaceae; [G] Microdochium;
62	KY430511	22	67608	Microdochium	Endophyte-Plant Pathogen	Pathotroph-Symbiotroph	Organic	O1	F	root; cellular organisms; [SK] Eukaryota; Opisthokonta; [K] Fungi; Dikarya; [P] Ascomycota; saccharomyceta; Pezizomycotina; leotiomyceta; sordariomyceta; [C] Sordariomycetes; Xylariomycetidae; [O] Xylariales; [F] Microdochiaceae; [G] Microdochium;
63	KY430512	19	252155	Schizothecium	Dung Saprotroph	Saprotroph	Conventional	C3	J	root; cellular organisms; [SK] Eukaryota; Opisthokonta; [K] Fungi; Dikarya; [P] Ascomycota; saccharomyceta; Pezizomycotina; leotiomyceta; sordariomyceta; [C] Sordariomycetes; Sordariomycetidae; [O] Sordariales; [F] Lasiosphaeriaceae; [G] Schizothecium;
64	KY430514	20	67608	Microdochium	Endophyte-Plant Pathogen	Pathotroph-Symbiotroph	Organic	O3	A	root; cellular organisms; [SK] Eukaryota; Opisthokonta; [K] Fungi; Dikarya; [P] Ascomycota; saccharomyceta; Pezizomycotina; leotiomyceta; sordariomyceta; [C] Sordariomycetes; Xylariomycetidae; [O] Xylariales; [F] Microdochiaceae; [G] Microdochium;
65	KY430515	9	231204	Leucosporidium golubevii	-	-	Organic	O3	I	root; cellular organisms; [SK] Eukaryota; Opisthokonta; [K] Fungi; Dikarya; [P] Basidiomycota; Pucciniomycotina; [C] Microbotryomycetes; [O] Leucosporidiales; [F] Leucosporidiaceae; [G] Leucosporidium; [S] Leucosporidium golubevii;
66	KY430516	20	67608	Microdochium	Endophyte-Plant Pathogen	Pathotroph-Symbiotroph	Organic	O3	H	root; cellular organisms; [SK] Eukaryota; Opisthokonta; [K] Fungi; Dikarya; [P] Ascomycota; saccharomyceta; Pezizomycotina; leotiomyceta; sordariomyceta; [C] Sordariomycetes; Xylariomycetidae; [O] Xylariales; [F] Microdochiaceae; [G] Microdochium;
67	KY430517	21	97971	Periconia	Endophyte-Plant Pathogen-Wood Saprotroph	Pathotroph-Saprotroph-Symbiotroph	Organic	O3	G	root; cellular organisms; [SK] Eukaryota; Opisthokonta; [K] Fungi; Dikarya; [P] Ascomycota; saccharomyceta; Pezizomycotina; leotiomyceta; dothideomyceta; [C] Dothideomycetes; Pleosporomycetidae; [O] Pleosporales; Massarineae; [F] Periconiaceae; [G] Periconia;
68	KY430518	7	4890	Ascomycota	-	-	Organic	O3	G	root; cellular organisms; [SK] Eukaryota; Opisthokonta; [K] Fungi; Dikarya; [P] Ascomycota;
69	KY430519	12	1577607	Slopeiomyces cylindrosporus	Plant Pathogen	Pathotroph	Organic	O2	I	root; cellular organisms; [SK] Eukaryota; Opisthokonta; [K] Fungi; Dikarya; [P] Ascomycota; saccharomyceta; Pezizomycotina; leotiomyceta; sordariomyceta; [C] Sordariomycetes; Sordariomycetidae; [O] Magnaporthales; [F] Magnaporthaceae; [G] Slopeiomyces; [S] Slopeiomyces cylindrosporus;
70	KY430520	20	67608	Microdochium	Endophyte-Plant Pathogen	Pathotroph-Symbiotroph	Organic	O2	H	root; cellular organisms; [SK] Eukaryota; Opisthokonta; [K] Fungi; Dikarya; [P] Ascomycota; saccharomyceta; Pezizomycotina; leotiomyceta; sordariomyceta; [C] Sordariomycetes; Xylariomycetidae; [O] Xylariales; [F] Microdochiaceae; [G] Microdochium;
71	KY430521	20	67608	Microdochium	Endophyte-Plant Pathogen	Pathotroph-Symbiotroph	Organic	O1	J	root; cellular organisms; [SK] Eukaryota; Opisthokonta; [K] Fungi; Dikarya; [P] Ascomycota; saccharomyceta; Pezizomycotina; leotiomyceta; sordariomyceta; [C] Sordariomycetes; Xylariomycetidae; [O] Xylariales; [F] Microdochiaceae; [G] Microdochium;
72	KY430522	20	67608	Microdochium	Endophyte-Plant Pathogen	Pathotroph-Symbiotroph	Conventional	C3	B	root; cellular organisms; [SK] Eukaryota; Opisthokonta; [K] Fungi; Dikarya; [P] Ascomycota; saccharomyceta; Pezizomycotina; leotiomyceta; sordariomyceta; [C] Sordariomycetes; Xylariomycetidae; [O] Xylariales; [F] Microdochiaceae; [G] Microdochium;
73	KY430523	20	67608	Microdochium	Endophyte-Plant Pathogen	Pathotroph-Symbiotroph	Conventional	C2	F	root; cellular organisms; [SK] Eukaryota; Opisthokonta; [K] Fungi; Dikarya; [P] Ascomycota; saccharomyceta; Pezizomycotina; leotiomyceta; sordariomyceta; [C] Sordariomycetes; Xylariomycetidae; [O] Xylariales; [F] Microdochiaceae; [G] Microdochium;
74	KY430524	20	67608	Microdochium	Endophyte-Plant Pathogen	Pathotroph-Symbiotroph	Conventional	C2	F	root; cellular organisms; [SK] Eukaryota; Opisthokonta; [K] Fungi; Dikarya; [P] Ascomycota; saccharomyceta; Pezizomycotina; leotiomyceta; sordariomyceta; [C] Sordariomycetes; Xylariomycetidae; [O] Xylariales; [F] Microdochiaceae; [G] Microdochium;
75	KY430525	20	67608	Microdochium	Endophyte-Plant Pathogen	Pathotroph-Symbiotroph	Conventional	C2	F	root; cellular organisms; [SK] Eukaryota; Opisthokonta; [K] Fungi; Dikarya; [P] Ascomycota; saccharomyceta; Pezizomycotina; leotiomyceta; sordariomyceta; [C] Sordariomycetes; Xylariomycetidae; [O] Xylariales; [F] Microdochiaceae; [G] Microdochium;
76	KY430526	20	67608	Microdochium	Endophyte-Plant Pathogen	Pathotroph-Symbiotroph	Conventional	C2	F	root; cellular organisms; [SK] Eukaryota; Opisthokonta; [K] Fungi; Dikarya; [P] Ascomycota; saccharomyceta; Pezizomycotina; leotiomyceta; sordariomyceta; [C] Sordariomycetes; Xylariomycetidae; [O] Xylariales; [F] Microdochiaceae; [G] Microdochium;
77	KY430527	3	68756	Clavulina	Ectomycorrhizal	Symbiotroph	Organic	O1	D	root; cellular organisms; [SK] Eukaryota; Opisthokonta; [K] Fungi; Dikarya; [P] Basidiomycota; Agaricomycotina; [C] Agaricomycetes; Agaricomycetes incertae sedis; [O] Cantharellales; [F] Clavulinaceae; [G] Clavulina;
78	KY430528	7	4890	Ascomycota	-	-	Organic	O3	G	root; cellular organisms; [SK] Eukaryota; Opisthokonta; [K] Fungi; Dikarya; [P] Ascomycota;
79	KY430529	7	4890	Ascomycota	-	-	Organic	O3	G	root; cellular organisms; [SK] Eukaryota; Opisthokonta; [K] Fungi; Dikarya; [P] Ascomycota;
80	KY430530	10	5277	Leucosporidium	-	-	Organic	O3	G	root; cellular organisms; [SK] Eukaryota; Opisthokonta; [K] Fungi; Dikarya; [P] Basidiomycota; Pucciniomycotina; [C] Microbotryomycetes; [O] Leucosporidiales; [F] Leucosporidiaceae; [G] Leucosporidium;
81	KY430531	26	61235	Fusarium equiseti	Animal Pathogen-Endophyte-Lichen Parasite-Plant Pathogen-Soil Saprotroph-Wood Saprotroph	Pathotroph-Saprotroph-Symbiotroph	Conventional	C1	B	root; cellular organisms; [SK] Eukaryota; Opisthokonta; [K] Fungi; Dikarya; [P] Ascomycota; saccharomyceta; Pezizomycotina; leotiomyceta; sordariomyceta; [C] Sordariomycetes; Hypocreomycetidae; [O] Hypocreales; [F] Nectriaceae; [G] Fusarium; Fusarium incarnatum-equiseti species complex; [S] Fusarium equiseti;
82	KY430532	32	343506	Podospora dimorpha	Dung Saprotroph-Endophyte-Litter Saprotroph-Undefined Saprotroph	Saprotroph-Symbiotroph	Organic	O1	F	root; cellular organisms; [SK] Eukaryota; Opisthokonta; [K] Fungi; Dikarya; [P] Ascomycota; saccharomyceta; Pezizomycotina; leotiomyceta; sordariomyceta; [C] Sordariomycetes; Sordariomycetidae; [O] Sordariales; [F] Lasiosphaeriaceae; [G] Podospora; [S] Podospora dimorpha;
83	KY430533	26	61235	Fusarium equiseti	Animal Pathogen-Endophyte-Lichen Parasite-Plant Pathogen-Soil Saprotroph-Wood Saprotroph	Pathotroph-Saprotroph-Symbiotroph	Conventional	C1	B	root; cellular organisms; [SK] Eukaryota; Opisthokonta; [K] Fungi; Dikarya; [P] Ascomycota; saccharomyceta; Pezizomycotina; leotiomyceta; sordariomyceta; [C] Sordariomycetes; Hypocreomycetidae; [O] Hypocreales; [F] Nectriaceae; [G] Fusarium; Fusarium incarnatum-equiseti species complex; [S] Fusarium equiseti;
84	KY430534	20	67608	Microdochium	Endophyte-Plant Pathogen	Pathotroph-Symbiotroph	Conventional	C3	G	root; cellular organisms; [SK] Eukaryota; Opisthokonta; [K] Fungi; Dikarya; [P] Ascomycota; saccharomyceta; Pezizomycotina; leotiomyceta; sordariomyceta; [C] Sordariomycetes; Xylariomycetidae; [O] Xylariales; [F] Microdochiaceae; [G] Microdochium;
85	KY430536	8	89926	Cystofilobasidium macerans	Leaf Saprotroph	Saprotroph	Organic	O1	H	root; cellular organisms; [SK] Eukaryota; Opisthokonta; [K] Fungi; Dikarya; [P] Basidiomycota; Agaricomycotina; [C] Tremellomycetes; [O] Cystofilobasidiales; [F] Cystofilobasidiaceae; [G] Cystofilobasidium; [S] Cystofilobasidium macerans;
86	KY430537	7	4890	Ascomycota	-	-	Organic	O1	G	root; cellular organisms; [SK] Eukaryota; Opisthokonta; [K] Fungi; Dikarya; [P] Ascomycota;
87	KY430539	7	4890	Ascomycota	-	-	Conventional	C4	B	root; cellular organisms; [SK] Eukaryota; Opisthokonta; [K] Fungi; Dikarya; [P] Ascomycota;
88	KY430540	22	67608	Microdochium	Endophyte-Plant Pathogen	Pathotroph-Symbiotroph	Conventional	C5	F	root; cellular organisms; [SK] Eukaryota; Opisthokonta; [K] Fungi; Dikarya; [P] Ascomycota; saccharomyceta; Pezizomycotina; leotiomyceta; sordariomyceta; [C] Sordariomycetes; Xylariomycetidae; [O] Xylariales; [F] Microdochiaceae; [G] Microdochium;
89	KY430541	20	67608	Microdochium	Endophyte-Plant Pathogen	Pathotroph-Symbiotroph	Conventional	C4	C	root; cellular organisms; [SK] Eukaryota; Opisthokonta; [K] Fungi; Dikarya; [P] Ascomycota; saccharomyceta; Pezizomycotina; leotiomyceta; sordariomyceta; [C] Sordariomycetes; Xylariomycetidae; [O] Xylariales; [F] Microdochiaceae; [G] Microdochium;
90	KY430542	7	4890	Ascomycota	-	-	Organic	O4	H	root; cellular organisms; [SK] Eukaryota; Opisthokonta; [K] Fungi; Dikarya; [P] Ascomycota;
91	KY430543	7	4890	Ascomycota	-	-	Organic	O4	B	root; cellular organisms; [SK] Eukaryota; Opisthokonta; [K] Fungi; Dikarya; [P] Ascomycota;
92	KY430544	7	4890	Ascomycota	-	-	Organic	O5	D	root; cellular organisms; [SK] Eukaryota; Opisthokonta; [K] Fungi; Dikarya; [P] Ascomycota;
93	KY430545	7	4890	Ascomycota	-	-	Organic	O4	A	root; cellular organisms; [SK] Eukaryota; Opisthokonta; [K] Fungi; Dikarya; [P] Ascomycota;
94	KY430546	7	4890	Ascomycota	-	-	Organic	O5	D	root; cellular organisms; [SK] Eukaryota; Opisthokonta; [K] Fungi; Dikarya; [P] Ascomycota;
95	KY430547	20	67608	Microdochium	Endophyte-Plant Pathogen	Pathotroph-Symbiotroph	Conventional	C5	H	root; cellular organisms; [SK] Eukaryota; Opisthokonta; [K] Fungi; Dikarya; [P] Ascomycota; saccharomyceta; Pezizomycotina; leotiomyceta; sordariomyceta; [C] Sordariomycetes; Xylariomycetidae; [O] Xylariales; [F] Microdochiaceae; [G] Microdochium;
96	KY430548	1	76775	Malassezia restricta	Animal Pathogen-Undefined Saprotroph	Pathotroph-Saprotroph	Conventional	C5	H	root; cellular organisms; [SK] Eukaryota; Opisthokonta; [K] Fungi; Dikarya; [P] Basidiomycota; Ustilaginomycotina; [C] Malasseziomycetes; [O] Malasseziales; [F] Malasseziaceae; [G] Malassezia; [S] Malassezia restricta;
97	KY430549	20	67608	Microdochium	Endophyte-Plant Pathogen	Pathotroph-Symbiotroph	Conventional	C5	E	root; cellular organisms; [SK] Eukaryota; Opisthokonta; [K] Fungi; Dikarya; [P] Ascomycota; saccharomyceta; Pezizomycotina; leotiomyceta; sordariomyceta; [C] Sordariomycetes; Xylariomycetidae; [O] Xylariales; [F] Microdochiaceae; [G] Microdochium;
98	KY430550	8	89926	Cystofilobasidium macerans	Leaf Saprotroph	Saprotroph	Conventional	C4	H	root; cellular organisms; [SK] Eukaryota; Opisthokonta; [K] Fungi; Dikarya; [P] Basidiomycota; Agaricomycotina; [C] Tremellomycetes; [O] Cystofilobasidiales; [F] Cystofilobasidiaceae; [G] Cystofilobasidium; [S] Cystofilobasidium macerans;
99	KY430551	20	67608	Microdochium	Endophyte-Plant Pathogen	Pathotroph-Symbiotroph	Conventional	C5	G	root; cellular organisms; [SK] Eukaryota; Opisthokonta; [K] Fungi; Dikarya; [P] Ascomycota; saccharomyceta; Pezizomycotina; leotiomyceta; sordariomyceta; [C] Sordariomycetes; Xylariomycetidae; [O] Xylariales; [F] Microdochiaceae; [G] Microdochium;
100	KY430552	25	4890	Ascomycota	-	-	Organic	O5	D	root; cellular organisms; [SK] Eukaryota; Opisthokonta; [K] Fungi; Dikarya; [P] Ascomycota;
101	KY430553	2	76775	Malassezia restricta	Animal Pathogen-Undefined Saprotroph	Pathotroph-Saprotroph	Conventional	C4	H	root; cellular organisms; [SK] Eukaryota; Opisthokonta; [K] Fungi; Dikarya; [P] Basidiomycota; Ustilaginomycotina; [C] Malasseziomycetes; [O] Malasseziales; [F] Malasseziaceae; [G] Malassezia; [S] Malassezia restricta;
102	KY430555	7	4890	Ascomycota	-	-	Organic	O5	D	root; cellular organisms; [SK] Eukaryota; Opisthokonta; [K] Fungi; Dikarya; [P] Ascomycota;
103	KY430556	22	67608	Microdochium	Endophyte-Plant Pathogen	Pathotroph-Symbiotroph	Conventional	C5	G	root; cellular organisms; [SK] Eukaryota; Opisthokonta; [K] Fungi; Dikarya; [P] Ascomycota; saccharomyceta; Pezizomycotina; leotiomyceta; sordariomyceta; [C] Sordariomycetes; Xylariomycetidae; [O] Xylariales; [F] Microdochiaceae; [G] Microdochium;
104	KY430557	22	67608	Microdochium	Endophyte-Plant Pathogen	Pathotroph-Symbiotroph	Conventional	C5	G	root; cellular organisms; [SK] Eukaryota; Opisthokonta; [K] Fungi; Dikarya; [P] Ascomycota; saccharomyceta; Pezizomycotina; leotiomyceta; sordariomyceta; [C] Sordariomycetes; Xylariomycetidae; [O] Xylariales; [F] Microdochiaceae; [G] Microdochium;
105	KY430558	7	4890	Ascomycota	-	-	Conventional	C5	A	root; cellular organisms; [SK] Eukaryota; Opisthokonta; [K] Fungi; Dikarya; [P] Ascomycota;
106	KY430559	7	4890	Ascomycota	-	-	Conventional	C5	A	root; cellular organisms; [SK] Eukaryota; Opisthokonta; [K] Fungi; Dikarya; [P] Ascomycota;
107	KY430560	4	5303	Polyporales	-	-	Conventional	C4	B	root; cellular organisms; [SK] Eukaryota; Opisthokonta; [K] Fungi; Dikarya; [P] Basidiomycota; Agaricomycotina; [C] Agaricomycetes; Agaricomycetes incertae sedis; [O] Polyporales;
108	KY430561	23	147548	Leotiomycetes	-	-	Conventional	C4	B	root; cellular organisms; [SK] Eukaryota; Opisthokonta; [K] Fungi; Dikarya; [P] Ascomycota; saccharomyceta; Pezizomycotina; leotiomyceta; sordariomyceta; [C] Leotiomycetes;
109	KY430563	20	67608	Microdochium	Endophyte-Plant Pathogen	Pathotroph-Symbiotroph	Conventional	C5	D	root; cellular organisms; [SK] Eukaryota; Opisthokonta; [K] Fungi; Dikarya; [P] Ascomycota; saccharomyceta; Pezizomycotina; leotiomyceta; sordariomyceta; [C] Sordariomycetes; Xylariomycetidae; [O] Xylariales; [F] Microdochiaceae; [G] Microdochium;
110	KY430564	20	67608	Microdochium	Endophyte-Plant Pathogen	Pathotroph-Symbiotroph	Conventional	C5	C	root; cellular organisms; [SK] Eukaryota; Opisthokonta; [K] Fungi; Dikarya; [P] Ascomycota; saccharomyceta; Pezizomycotina; leotiomyceta; sordariomyceta; [C] Sordariomycetes; Xylariomycetidae; [O] Xylariales; [F] Microdochiaceae; [G] Microdochium;
111	KY430565	7	4890	Ascomycota	-	-	Conventional	C4	B	root; cellular organisms; [SK] Eukaryota; Opisthokonta; [K] Fungi; Dikarya; [P] Ascomycota;
112	KY430566	7	4890	Ascomycota	-	-	Conventional	C4	B	root; cellular organisms; [SK] Eukaryota; Opisthokonta; [K] Fungi; Dikarya; [P] Ascomycota;
113	KY430567	7	4890	Ascomycota	-	-	Conventional	C4	B	root; cellular organisms; [SK] Eukaryota; Opisthokonta; [K] Fungi; Dikarya; [P] Ascomycota;
114	KY430568	7	4890	Ascomycota	-	-	Conventional	C5	A	root; cellular organisms; [SK] Eukaryota; Opisthokonta; [K] Fungi; Dikarya; [P] Ascomycota;
115	KY430569	24	147548	Leotiomycetes	-	-	Conventional	C5	A	root; cellular organisms; [SK] Eukaryota; Opisthokonta; [K] Fungi; Dikarya; [P] Ascomycota; saccharomyceta; Pezizomycotina; leotiomyceta; sordariomyceta; [C] Leotiomycetes;
116	KY430570	20	67608	Microdochium	Endophyte-Plant Pathogen	Pathotroph-Symbiotroph	Conventional	C5	C	root; cellular organisms; [SK] Eukaryota; Opisthokonta; [K] Fungi; Dikarya; [P] Ascomycota; saccharomyceta; Pezizomycotina; leotiomyceta; sordariomyceta; [C] Sordariomycetes; Xylariomycetidae; [O] Xylariales; [F] Microdochiaceae; [G] Microdochium;
117	KY430571	37	4972	Dioszegia hungarica	Fungal Parasite-Undefined Saprotroph	Pathotroph-Saprotroph-Symbiotroph	Conventional	C5	D	root; cellular organisms; [SK] Eukaryota; Opisthokonta; [K] Fungi; Dikarya; [P] Basidiomycota; Agaricomycotina; [C] Tremellomycetes; [O] Tremellales; [F] Bulleribasidiaceae; [G] Dioszegia; [S] Dioszegia hungarica;
118	KY430572	20	67608	Microdochium	Endophyte-Plant Pathogen	Pathotroph-Symbiotroph	Conventional	C5	D	root; cellular organisms; [SK] Eukaryota; Opisthokonta; [K] Fungi; Dikarya; [P] Ascomycota; saccharomyceta; Pezizomycotina; leotiomyceta; sordariomyceta; [C] Sordariomycetes; Xylariomycetidae; [O] Xylariales; [F] Microdochiaceae; [G] Microdochium;
119	KY430573	35	1895944	Vishniacozyma victoriae	Fungal Parasite-Undefined Saprotroph	Pathotroph-Saprotroph-Symbiotroph	Conventional	C5	D	root; cellular organisms; [SK] Eukaryota; Opisthokonta; [K] Fungi; Dikarya; [P] Basidiomycota; Agaricomycotina; [C] Tremellomycetes; [O] Tremellales; [F] Bulleribasidiaceae; [G] Vishniacozyma; [S] Vishniacozyma victoriae;
120	KY430574	17	983939	Holtermanniella	-	-	Conventional	C5	D	root; cellular organisms; [SK] Eukaryota; Opisthokonta; [K] Fungi; Dikarya; [P] Basidiomycota; Agaricomycotina; [C] Tremellomycetes; [O] Holtermanniales; [G] Holtermanniella;
121	KY430575	20	67608	Microdochium	Endophyte-Plant Pathogen	Pathotroph-Symbiotroph	Conventional	C5	E	root; cellular organisms; [SK] Eukaryota; Opisthokonta; [K] Fungi; Dikarya; [P] Ascomycota; saccharomyceta; Pezizomycotina; leotiomyceta; sordariomyceta; [C] Sordariomycetes; Xylariomycetidae; [O] Xylariales; [F] Microdochiaceae; [G] Microdochium;
122	KY430576	20	67608	Microdochium	Endophyte-Plant Pathogen	Pathotroph-Symbiotroph	Conventional	C5	G	root; cellular organisms; [SK] Eukaryota; Opisthokonta; [K] Fungi; Dikarya; [P] Ascomycota; saccharomyceta; Pezizomycotina; leotiomyceta; sordariomyceta; [C] Sordariomycetes; Xylariomycetidae; [O] Xylariales; [F] Microdochiaceae; [G] Microdochium;
123	KY430577	20	67608	Microdochium	Endophyte-Plant Pathogen	Pathotroph-Symbiotroph	Conventional	C5	G	root; cellular organisms; [SK] Eukaryota; Opisthokonta; [K] Fungi; Dikarya; [P] Ascomycota; saccharomyceta; Pezizomycotina; leotiomyceta; sordariomyceta; [C] Sordariomycetes; Xylariomycetidae; [O] Xylariales; [F] Microdochiaceae; [G] Microdochium;
124	KY430578	24	147548	Leotiomycetes	-	-	Conventional	C5	G	root; cellular organisms; [SK] Eukaryota; Opisthokonta; [K] Fungi; Dikarya; [P] Ascomycota; saccharomyceta; Pezizomycotina; leotiomyceta; sordariomyceta; [C] Leotiomycetes;
125	KY430579	1	55193	Malassezia	Animal Pathogen-Undefined Saprotroph	Pathotroph-Saprotroph	Conventional	C5	H	root; cellular organisms; [SK] Eukaryota; Opisthokonta; [K] Fungi; Dikarya; [P] Basidiomycota; Ustilaginomycotina; [C] Malasseziomycetes; [O] Malasseziales; [F] Malasseziaceae; [G] Malassezia;
126	KY430580	0	155619	Agaricomycetes	-	-	Conventional	C5	J	root; cellular organisms; [SK] Eukaryota; Opisthokonta; [K] Fungi; Dikarya; [P] Basidiomycota; Agaricomycotina; [C] Agaricomycetes;
127	KY430581	18	457084	Fusarium nurragi	Animal Pathogen-Endophyte-Lichen Parasite-Plant Pathogen-Soil Saprotroph-Wood Saprotroph	Pathotroph-Saprotroph-Symbiotroph	Conventional	C5	J	root; cellular organisms; [SK] Eukaryota; Opisthokonta; [K] Fungi; Dikarya; [P] Ascomycota; saccharomyceta; Pezizomycotina; leotiomyceta; sordariomyceta; [C] Sordariomycetes; Hypocreomycetidae; [O] Hypocreales; [F] Nectriaceae; [G] Fusarium; [S] Fusarium nurragi;
128	KY430582	0	155619	Agaricomycetes	-	-	Conventional	C5	J	root; cellular organisms; [SK] Eukaryota; Opisthokonta; [K] Fungi; Dikarya; [P] Basidiomycota; Agaricomycotina; [C] Agaricomycetes;
129	KY430583	0	155619	Agaricomycetes	-	-	Conventional	C5	J	root; cellular organisms; [SK] Eukaryota; Opisthokonta; [K] Fungi; Dikarya; [P] Basidiomycota; Agaricomycotina; [C] Agaricomycetes;
130	KY430584	16	437017	Paraphoma radicina	Fungal Parasite-Plant Pathogen-Plant Saprotroph	Pathotroph-Saprotroph	Organic	O2	H	root; cellular organisms; [SK] Eukaryota; Opisthokonta; [K] Fungi; Dikarya; [P] Ascomycota; saccharomyceta; Pezizomycotina; leotiomyceta; dothideomyceta; [C] Dothideomycetes; Pleosporomycetidae; [O] Pleosporales; Pleosporineae; [F] Phaeosphaeriaceae; [G] Paraphoma; [S] Paraphoma radicina;
